# Effects of a forming process on the properties and structure of RANEY®-Ni catalysts for the hydrogenation of 1,4-butenediol

**DOI:** 10.1039/c9ra10200k

**Published:** 2020-02-05

**Authors:** Xianlong Gao, Wenlong Mo, Fengyun Ma, Tsubaki Noritatsu, Hongli Wu, Xing Fan

**Affiliations:** Key Laboratory of Coal Clean Conversion & Chemical Engineering Process (Xinjiang Uyghur Autonomous Region), College of Chemistry and Chemical Engineering, Xinjiang University Urumqi Xinjiang 830046 China mowenlong@xju.edu.cn ma_fy@126.com; Department of Applied Chemistry, School of Engineering, University of Toyama Gofuku 3190 Toyama 930-8555 Japan

## Abstract

Three commercial Ni–Al alloys formed by a vacuum atomization method (NAV), atmospheric atomization method (NAA) and high-temperature melting method (NAH) were leached by 10 wt% NaOH solution to prepare three RANEY®-Ni catalysts (RNAV, RNAA and RNAH, correspondingly). The effects of a forming process on the structure of Ni–Al alloys and the corresponding RANEY®-Ni catalysts were investigated *via* XRD, XPS, SEM, TEM, NH_3_-TPD, N_2_ adsorption–desorption and EDX-mapping studies. Also, the as-prepared RANEY®-Ni catalysts were evaluated *via* the hydrogenation of 1,4-butenediol (BED) to produce 1,4-butanediol (BDO). The results showed that the specific surface areas and surface morphologies of the Ni–Al alloys present significant differences. Meanwhile, the RNAA sample presented a comparatively regular morphology, similar to a small piece of sugar cane. The weak and medium acid peak areas of the RNAA catalyst were lower than those of the other samples. RNAV showed higher weak and medium acid peak areas, demonstrating the higher number of acid centers on the surface of the catalyst. The surface of the RNAA catalyst obtained from NAA contained more active component-Ni, about 90 wt% on the surface, and the specific surface area of the sample was 75 times that of its precursor Ni–Al alloy powder (NAA). The evaluation results present that the RNAA catalyst shows better hydrogenation performance, with BED conversion of 100%, both BDO selectivity and yield of 46.11%.

## Abstract 2

Xianlong Gao's paper focuses on the effects of a forming process on the catalytic hydrogenation of 1,4-butenediol (BED) to produce 1,4-butanediol (BDO). The evaluation results showed that the RNAA catalyst showed excellent hydrogenation performance.

## Graphical abstract

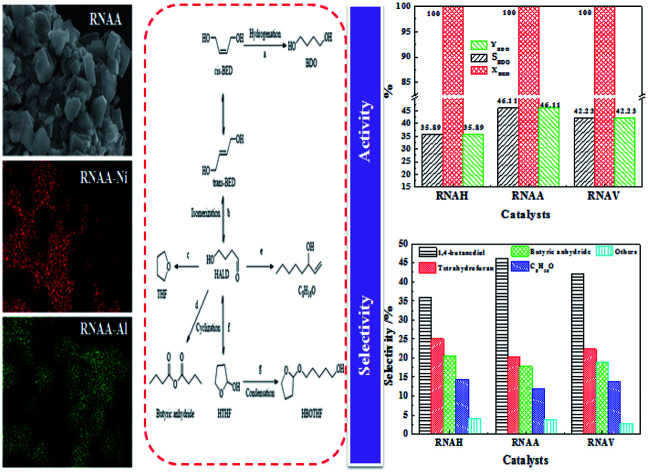

## Introduction

Selective hydrogenation of carbon–carbon double bonds to single bonds is important to produce valuable or fine chemicals. 1,4-Butanediol (BDO) is one of the most important organic and fine chemical raw materials; its derivatives are widely used in medicine, fiber and engineering plastics.^1–6^ The main process of BDO production is the Reppe method, which includes two steps;^7,8^ the production of 1,4-butynediol (BYD) from acetylene and formaldehyde and the hydrogenation of 1,4-butenediol (BED) to prepare 1,4-butanediol occur by two sequential processes, namely BYD to BED and BED to BDO. However, depth hydrogenation of BED is accompanied by side reactions, including double migration, hydrogenolysis, and isomerization, to form 4-hydroxybutanal (HALD), tetrahydrofuran, 1-ene-3-octanol, 2-hydroxytetrahydrofuran (HTHF), and 2-(4-hydroxybutoxy)-tetrahydrofuran (HBOTHF) by-products. Therefore, tuneable hydrogenation ability of BED to BDO is a crucial method for industrial use. The most commonly used catalyst is RANEY®-Ni. However, there are few reports considering catalysts prepared from alloys for BED hydrogenation.

Zhang *et al.*^9^ adopted low pressure plasma spray (LPPS), high velocity oxygen fuel (HVOF) and low temperature high velocity oxygen fuel (LT-HVOF) to prepare Ni–Cr–Al–Y alloys on nickel-base superalloy K438 substrate. The results, from the standpoints of microstructure, element distribution, phase composition and basic properties, showed that the LPPS alloy presented a dense layered structure with high microhardness and good oxidation resistance; meanwhile, the HVOF alloy showed a typical layered structure with low microhardness and poor oxidation resistance, and the LT-HVOF alloy presented greatly decreased microhardness and good oxidation resistance. Cui *et al.*^10^ prepared Ag–25Ni alloy by powder metallurgy (PM), mechanical alloying (MA) and liquid phase reduction (LPR) methods. The electrochemical corrosion properties of the three alloys in 0.3 mol L^−1^ sodium chloride solution were investigated. The results showed that the corrosion current density of Ag–25Ni alloys decreased in the order of LPR Ag–25Ni, PM Ag–25Ni and MA Ag–25Ni. The passivation films formed by the three alloys were ascribed to n-type semiconductors; also, the passivation performance and chemical stability of MA Ag–25Ni alloy were better, which was attributed to the small grain size of MA Ag–25Ni alloy and the good solid solubility between Ag and Ni. Sun *et al.*^11^ carried out ultrasonic testing on FGH4096 alloys derived from different forming processes (hot isostatic pressing + forging and direct hot isostatic pressing). The results showed that the alloy obtained from the hot isostatic pressing + forging process demonstrated obvious defect signals, while the direct hot isostatic pressing alloy presented no defect signals. Therefore, the physical and chemical properties of materials obtained from various molding processes showed great differences.

There are many reports on the preparation process of RANEY®-Ni catalyst precursor (Ni–Al alloy).^12,13^ Wang *et al.*^14^ treated Ni–Al alloy with a high energy ball-milling machine and leached it with NaOH solution to produce RANEY®-Ni catalyst. The crystal structures and surface morphologies of the Ni–Al alloy and the corresponding RANEY®-Ni catalyst changed greatly compared to those of the untreated sample. The performance of the catalyst was investigated by hydrogenation of cyclohexanone. The results showed that the content of NiAl_3_ species in the Ni–Al alloys decreased with increasing ball milling time, while the content of Ni_2_Al_3_ crystal increased gradually. The treated RANEY®-Ni catalyst with a ball milling time of 12 h demonstrated better hydrogenation performance, with cyclohexanone conversion of 3.8%. Lee *et al.*^15^ investigated the structures of RANEY®-Ni catalysts prepared from Ni–Al alloys with different Ni contents (42 wt%, 50 wt% and 60 wt%). XRD characterization showed that the samples exhibited different phases with increasing Ni content. Ni42 and Ni50 were mainly composed of three phases of NiAl_3_, Ni_2_Al_3_ and eutectic Al. Ni50 contained more Ni_2_Al_3_ crystal and a small amount of NiAl_3_ phase, while Ni60 presented only Ni_2_Al_3_ phase. This study signified that Ni_2_Al_3_ phase demonstrated higher strength and NiAl_3_ phase showed better activity.^16^ The preparation of RANEY®-Ni catalysts with both good strength and activity is worthy of further study.

In the traditional BED hydrogenation process, noble metal (Ru,^17^ Pt,^18^ Pd,^19^*etc.*) catalysts exhibit high catalytic activity. However, noble metals are scarce and expensive, which limits their large-scale application in industry. In order to meet the industrial demand, it is necessary to develop economical catalysts, such as RANEY®-Ni. It is reported that skeleton structure catalysts (catalysts prepared from alloys) can display superior catalytic properties (*e.g.* activity, selectivity and stability) during the hydrogenation process to those of nickel-based catalysts.^20,21^ Ni–Al alloys from different forming processes were leached by NaOH solution (vacuum atomization method, atmospheric atomization method and high temperature melting method) to prepare corresponding RANEY®-Ni catalysts, which were characterized by EDX, XRD N_2_-adsorption desorption, XPS, TEM and NH_3_-TPD methods. The effects of the different forming processes on the structure of Ni–Al alloy and the corresponding RANEY®-Ni catalyst were studied to achieve an optimized catalyst design and reach a maximum efficiency for the catalytic hydrogenation of 1,4-butenediol to 1,4-butanediol.

## Experimental

### Material synthesis

Ni–Al alloys (100 to 120 mesh) produced by different forming processes (vacuum atomization method, atmospheric atomization method (both from Qinghe County Aerospace Metal Materials Co., Ltd., China) and high-temperature melting method (Liaoning Zhongli Catalysts Technology Co., Ltd., China)) were leached by 10 wt% NaOH solution and stirred in a water bath at 80 °C for 90 min. The mixture was washed with absolute ethanol several times until the washing solution was neutral, then finally stored in absolute ethanol; thus, the RANEY®-Ni catalyst was obtained. The prepared RANEY®-Ni catalysts were named RNAA, RNAH and RNAV according to their forming processes.

### Material characterization

X-ray diffraction (XRD) analysis was carried out on an X-ray diffraction device (Rigaku D/Max-2500, Japan) using nickel filtered Cu Kα (*λ* = 0.15406 nm) radiation. The scan rate, diffraction range, tube voltage and tube current were 8° min^−1^, 5° to 80°, 40 kV and 100 mA, respectively. Nitrogen adsorption–desorption profiles at −196 °C were obtained by a Quantachrome Automated Gas Sorption apparatus (Micromeritics ASAP 2020). X-ray energy dispersive (EDX) analysis was carried out on a LEO 1530VP spectrometer from Germany with an accelerating voltage of 20 kV, working distance of 15 mm and acquisition time of 120 s. Scanning electron microscopy (SEM) images were obtained on a Hitachi H-600 microscope with an accelerated voltage of 100 kV. Transmission electron microscopy (TEM) micrographs were obtained using a JEOL JEM-2100 election microscope operating at 200 kV. The acidic properties of the catalysts were measured *via* temperature-programmed desorption of ammonia (NH_3_-TPD) using a Quantachrome Chemisorb instrument. A Thermo Fisher Scientific instrument (USA) was used for the X-ray photoelectron spectroscopy (XPS). The model is ESCALAB 250Xi, and the X-ray source was a monochromator, Al K alpha 1486.6 eV.

### Catalytic performance

**Fig. 1:**
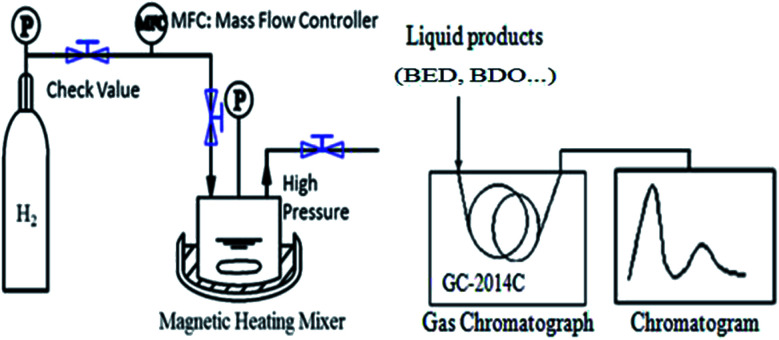
Scheme of the evaluation device for 1,4-butenediol hydrogenation.

BED hydrogenation was conducted in a 50 mL high-pressure reactor (Dalian Tongda reactor factory, CJF-605, China), and the products were analyzed by a GC (GC 2014, Shimadzu Japan) equipped with an FID detector and an SH-RtxWax capillary column (30 m × 0.53 mm i.d.). The quantitative method for the GC analysis was calibrated with 1,3-propanediol as an internal standard. Fig. 1 shows a schematic of the hydrogenation and product analysis system setup. BED hydrogenation was carried out at 393 K under 5.0 MPa for 3 h, with a stirring rate of 400 rpm and catalyst addition of 0.4 g. The feedstock was 30 mL aqueous solution containing 35 wt% 1,4-butenediol.

1,4-Butanediol, 1,4-butenediol, butyric anhydride, C_8_H_16_O and tetrahydrofuran were the main substances in the catalytic hydrogenation product of 1,4-butenediol; these were detected by a SH-Rtx-Wax capillary column. The performance of a hydrogenation catalyst is evaluated by its BED conversion, BDO selectivity and yield, which can be calculated by eqn (1), (2) and (3), respectively.

Performance of the catalyst:

1 BED conversion, *X* (%) = 1 − (*C*_2_/(*C*_1_ + *C*_2_ + *C*_3_ + *C*_4_ + *C*_5_)) × 100

2 BDO selectivity, *S* (%) = *C*_1_/(*C*_1_ + *C*_3_ + *C*_4_ + *C*_5_) × 100

3 BDO yield, *Y* (%) = *X* × *S* × 100

*C*_*i*_ represents the molar percentage of the corresponding substance. *i* = 1, 2, 3, 4 and 5 represent the components 1,4-butanediol, 1,4-butenediol, butyric anhydride, tetrahydrofuran, and C_8_H_16_O, respectively.

## Results and discussion

### Textural characteristics

**Fig. 2:**
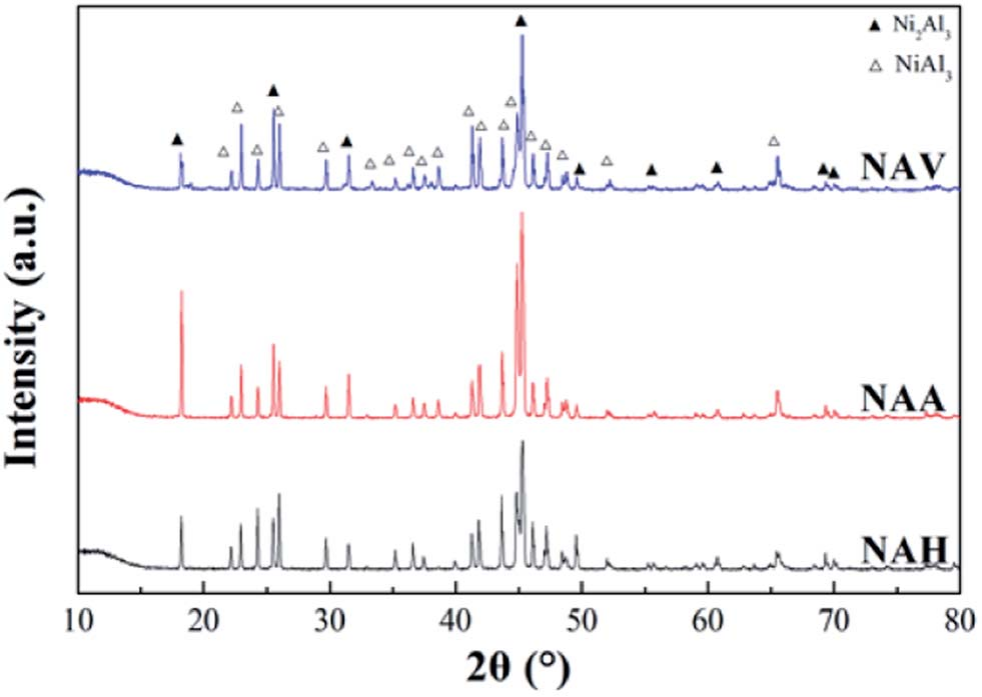
XRD patterns of the Ni–Al alloys.

Fig. 2 shows the XRD patterns of the Ni–Al alloys obtained from different forming methods. All the samples were composed of two crystal phases of NiAl_3_ and Ni_2_Al_3_.^22,23^ The diffraction peak positions of NiAl_3_ and Ni_2_Al_3_ for the three samples were the same, indicating that the forming method did not change the crystal composition of the Ni–Al alloy. Meanwhile, the diffraction peak intensities and half-peak widths of the two crystals were quite different, demonstrating that the grain sizes of the two species were different. NAA produced by the atmospheric atomization method demonstrated a high diffraction peak intensity and low half-peak width of NiAl_3_ phase and Ni_2_Al_3_ phase, indicating that its crystal structure was more complete. According to Scherrer's law (*D* = 0.89*λ*/(*β* cos *θ*)), the grain sizes of Ni_2_Al_3_ and NiAl_3_ were calculated at 2*θ* = 18.2° and 2*θ* = 24.5°, and the results are shown in Table 1.

**Table 1:** Crystal grain sizes of Ni_2_Al_3_ and NiAl_3_^a^

Sample	Size of Ni_2_Al_3_ (nm)	Size of NiAl_3_ (nm)
NAV	1.05	1.29
NAA	1.49	1.16
NAH	1.23	1.56

a Calculated by the Scherrer formula at the 2 theta angles of Ni_2_Al_3_ and NiAl_3_.

**Fig. 3:**
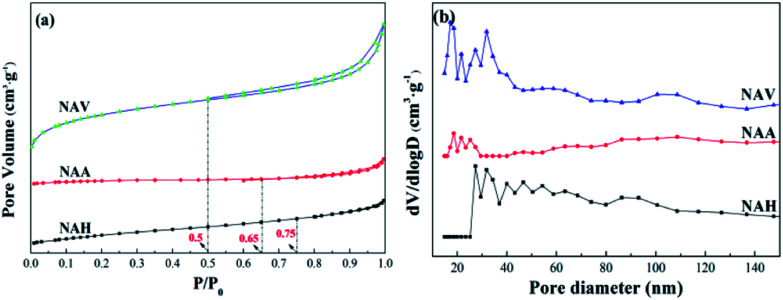
N_2_ adsorption–desorption isotherms (a) and pore distributions (b) of the alloys.

The N_2_ adsorption–desorption characterization results are shown in Fig. 3. According to the International Union of Theoretical and Applied Chemistry (IUPAC) classification, it can be seen that the adsorption isotherms can be ascribed to type III, which is a feature of weak gas–solid interactions on a solid surface. The corresponding hysteresis loops are type H3, and the morphology may be a sheet structure.^24^

**Fig. 4:**
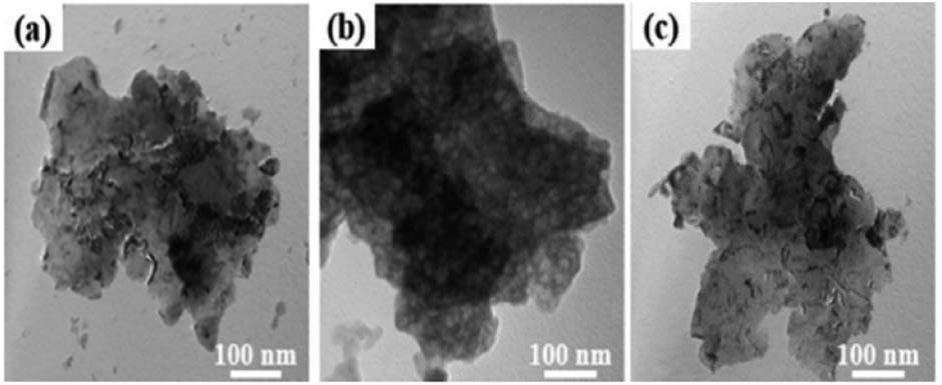
TEM images of the Ni–Al alloys: (a) NAV; (b) CNAA; (c) NAH.

Fig. 3(a) depicts the N_2_ adsorption–desorption isotherms of the Ni–Al alloys. The three samples present large differences in the areas of their hysteresis loops. The hysteresis loops of NAA and NAH are extremely small, and their isothermal adsorption lines and desorption lines nearly coincide. Also, the three samples show large differences in their adsorption and desorption curve closure points, with NAH closing at *P*/*P*_0_ = 0.75, NAA at *P*/*P*_0_ = 0.65 and NAV at *P*/*P*_0_ = 0.5. The pore structures of the samples are quite different; NAH and NAA contain more mesopores,^25^ while NAV showed a certain number of macropores,^26^ which is consistent with the pore size distributions shown in Fig. 4(b).

Fig. 3(b) shows the pore size distributions of the three samples obtained from the different forming methods. According to the principle of N_2_ adsorption–desorption isotherms, a high d*V*/dlog *D* value indicates more pores in the corresponding pore size range. The pore diameter peaks of all the samples were in the range of 10 to 50 nm, demonstrating that the three samples are mesoporous materials.^27^ The most probable pore diameters of the samples were quite different; those of the NAV and NAH samples were about 30 nm, while that of NAA was nearly 20 nm.

According to the N_2_ adsorption–desorption isotherms, the specific surface areas of the samples were calculated by the BET equation and the pore volumes and average pore diameters were calculated by the BJH equation, as shown in Table 2. The average pore sizes of NAV and NAH were about 6.0 nm, and that of NAA was about 13.4 nm; these are not consistent with the results of the most probable pore sizes in the N_2_ adsorption–desorption curves (Fig. 3(b)). Because the pore structure parameters were calculated from the microporous portions of the materials, the N_2_ physical adsorption results show only the mesoporous sizes. Thus, the pore size distribution only includes pores greater than 10 nm, and no microporous pores were included. The specific surface areas of all the samples also displayed large differences, with 0.86 m^2^ g^−1^ for NAA and 12.15 m^2^ g^−1^ for NAV; this may be due to the fact that the sample used in the vacuum atomization method was a liquid metal or alloy with gas supersaturation during the preparation process. The gas can expand to transform the liquid metal or alloy into powder under vacuum conditions; this forms a large amount of pores, resulting in a larger specific surface area.^28,29^ The melting process fused the metals, nickel and aluminum, in the melting furnace; the obtained melt was quenched and cooled, then pulverized into uniform fine particles. The atmospheric atomization method uses a high-speed air stream or liquid stream to directly crush the liquid metal or alloy to obtain metal powder; this process involves almost no pore formation and presents low specific surface areas.^30^

**Table 2:** Specific surface areas, pore volumes, and average pore diameters of the alloy powder

Samples	Specific surface area^a^ (m^2^ g^−1^)	Pore volume^b^ (cm^3^ g^−1^)	Average pore diameter^c^ (nm)
NAV	12.15	0.011	5.66
NAA	0.86	0.002	13.39
NAH	2.78	0.003	5.86

a Calculated by the Brunauer–Emmett–Teller (BET) equation.

b Barrett–Joyner–Halenda (BJH) desorption average pore diameter.

c Barrett–Joyner–Halenda (BJH) desorption pore volume.

Fig. 4 shows TEM images of the Ni–Al alloys at a magnification of 100 000. All the samples showed a sheet morphology. The alloy produced by the vacuum atomization method and high-temperature melting process presented a petal-like morphology. For the NAA sample, the porous surface structure can be clearly observed, which may be related to its crystal phase properties (composed of Ni_2_Al_3_, NiAl_3_, Ni_3_Al, *etc.*).^31,32^

**Fig. 5:**
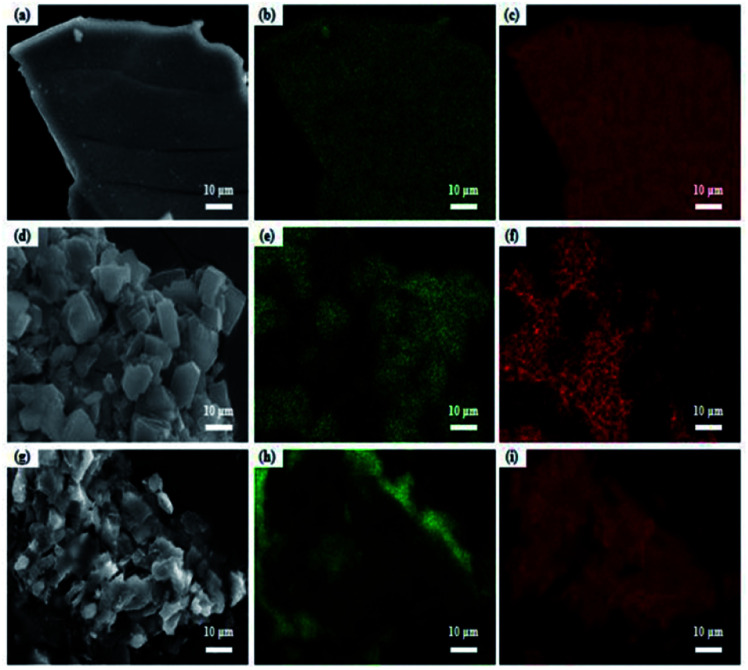
EDX-mapping photos of the catalysts: (a) RNAV; (b) RNAV-Al; (c) RNAV-Ni; (d) RNAA; (e) RNAA-Al; (f) RNAA-Ni; (g) RNAH; (h) RNAH-Al; (i) RNAH-Ni.

EDX analysis was carried out for the prepared catalysts, and the results are given in Table 3. It can be seen from Table 3 that the relative Ni content of each sample was more than 87 wt%, which was 1.81 times higher than the Ni content (48 wt%) in the alloy powder; this indicates that a large amount of Al element was washed away during the leaching process (① Al + OH^−^ → [Al(OH)_4_]^−^, ② [Al(OH)_4_]^−^ → Al(OH)_3_ + OH^−^, with the production of aluminum hydroxide and hydrated alumina). Some aluminum was also retained. Once Al element was completely leached, the skeleton structure of the RANEY®-Ni catalyst was seriously damaged, resulting in crystal agglomeration and decreasing the activity of the catalyst.^33,34^ More Ni species on the RNAA catalyst were detected with a content of 90 wt%, showing that it possesses more active sites. Additionally, the atomic ratio of Ni/Al of each sample is shown in Table 3. It can be seen from Table 3 that the RNAA sample presented a large Ni/Al molar ratio, which may be related to a certain degree of agglomeration of Ni (Fig. 5). The ratio of RNAA was large (3.93), which may be derived from the better dispersion of Ni on the surface of the catalyst (Fig. 5).

**Table 3:** Contents of elements in the samples

Sample	Al/wt%	Ni/wt%	*N*(Ni)/*n*(Al)
RNAH	12.94	87.06	3.09
RNAA	10.47	89.53	3.93
RNAV	11.48	88.52	3.55

To verify the distribution of each element on the RANEY®-Ni surface, EDX-mapping tests were performed, and the results are shown in Fig. 5. It can be seen from the mapping diagram that Ni and Al are distributed uniformly, and the Al element on the surface of RNAV was relatively sparse compared to the distribution of Ni element.^35^

**Fig. 6:**
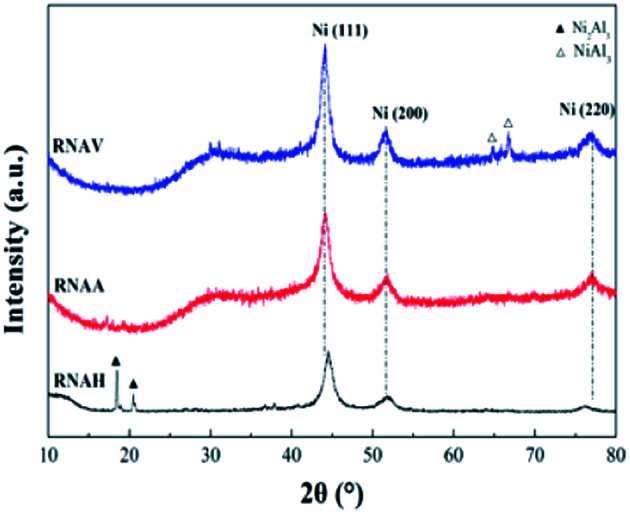
XRD patterns of the catalysts.

Fig. 6 shows the XRD patterns of the three RANEY®-Ni catalysts. All the catalysts presented characteristic diffraction peaks at 2*θ* = 44.1°, 51.3° and 76.3°, corresponding to Ni(111), Ni(200) and Ni(220), respectively.^36^ There were no Ni_2_Al_3_ or NiAl_3_ diffraction peaks for the RNAA sample, indicating that the leaching process was relatively complete (with a Ni/Al ratio as high as 3.93). For the RNAH sample, the diffraction peaks of Ni_2_Al_3_ species were observed at 2*θ* = 18.4° and 20.5°. Also, the diffraction peaks of the NiAl_3_ species were detected in RNAV at 2*θ* = 65.0° and 66.7°.

For RNAH, the diffraction peak intensity of Ni was lower than those of RNAV and RNAA, and the half-peak width (*β*) was larger. According to Scherrer's formula (*D* = 0.89*λ*/(*β* cos *θ*)), it is obvious that the Ni crystal in RNAH is small in size.

According to Scherrer's law (*D* = 0.89*λ*/(*β* cos *θ*)), the grain sizes of Ni crystal were calculated at 2*θ* = 44.1°, and the results are shown in Table 4.

**Table 4:** Ni grain sizes of the catalysts before and after hydrogenation^a^

Sample	Before reaction with Ni (nm)	After reaction with Ni (nm)
RNAH	0.136	0.088
RNAA	0.166	0.097
RNAV	0.170	0.116

a Calculated by the Scherrer formula at the 2 theta angle of Ni(111).

**Fig. 7:**
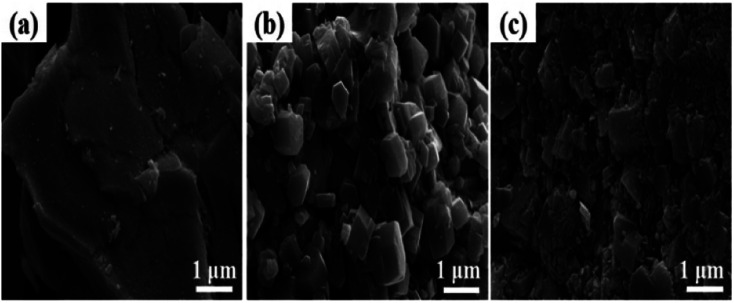
SEM images of the catalysts: (a) RNAV; (b) RNAA; (c) RNAH.

Fig. 7 shows the SEM images of the catalysts. Relatively fragmented, irregularly shaped particles can be clearly observed on the surface of the RNAH catalyst. Meanwhile, the RNAA sample presents a comparatively regular morphology, similar to a small piece of sugar cane.^30^ In addition, there are some finer particles on the surface of the RNAV sample.

**Fig. 8:**
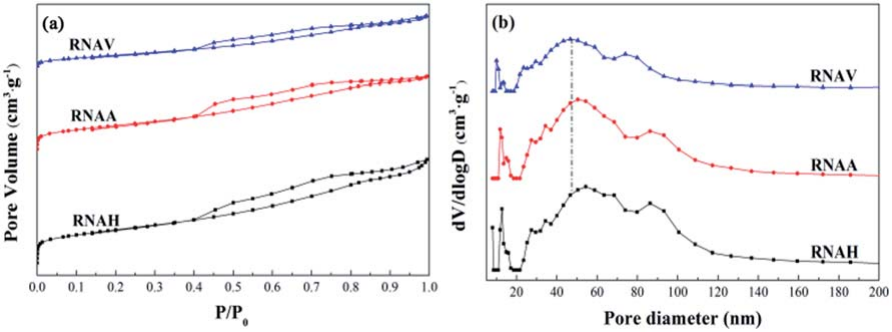
N_2_ adsorption–desorption isotherms (a) and pore distributions (b) of the catalysts.

Fig. 8 shows the N_2_ adsorption–desorption isotherms (a) and pore size distributions (b) of the prepared catalysts. According to the IUPAC classification, the isothermal adsorption lines of the three catalysts can be ascribed to type III, indicating that the adsorption heat of N_2_ on the catalyst is lower than its liquefaction heat. Also, the adsorption isotherm type of each sample was almost the same, and there are obvious H3 hysteresis loops in the range of *P*/*P*_0_ = 0.4 to 1.0.

Fig. 8(a) shows that the area of the hysteresis loop is different for each catalyst. Also, the hysteresis loop area of RNAH is larger than that of RNAV. Fig. 8(b) describes the pore size distributions of the prepared catalysts. Compared with the pore size distribution of Ni–Al alloy, the most probable pore size varied significantly. Because of the leaching process with NaOH solution, the pore size of each sample showed three-stage distribution characteristics of 10 to 20 nm, 40 to 70 nm and 80 to 100 nm, which is a typical feature of mesoporous–mesoporous–macroporous multi-level pore distribution.

The specific surface areas of the prepared catalysts were calculated by the BET equation, and the pore volumes and average pore diameters were calculated by the BJH equation, as shown in Table 5. The areas of the three samples were between 35 m^2^ g^−1^ and 85 m^2^ g^−1^, the pore volumes were 0.05 to 0.10 cm^3^ g^−1^, and the average pore diameters were 4 to 6 nm, indicating that the prepared catalysts are mesoporous materials. Compared with the corresponding Ni–Al alloy, the surface area of RNAA increased greatly, to 75 times that of NAA. This may be because the surface of the RNAA sample presented a relatively porous structure (TEM), and the average pore size changed greatly from 13.39 nm (NAA) to 4.29 nm.

**Table 5:** Specific surface areas, pore volumes, and average pore diameters of the catalysts

Sample	Specific surface area^a^ (m^2^ g^−1^)	Pore volume^b^ (cm^3^ g^−1^)	Average pore diameter^c^ (nm)
RNAV	37.43	0.05	4.90
RNAA	65.50	0.07	4.29
RNAH	84.36	0.10	5.17

a Calculated by the Brunauer–Emmett–Teller (BET) equation.

b Barrett–Joyner–Halenda (BJH) desorption average pore diameter.

c Barrett–Joyner–Halenda (BJH) desorption pore volume.

**Fig. 9:**
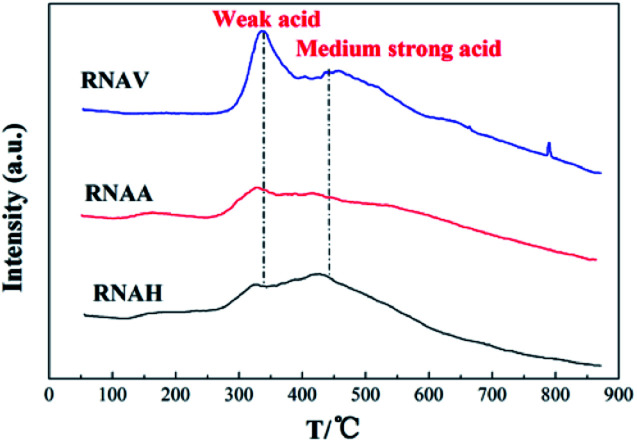
NH_3_-TPD profiles of the catalysts.

Fig. 9 shows the NH_3_-TPD diagrams of the catalysts. It was observed that all the samples presented two distinct NH_3_ desorption peaks around 330 °C and 450 °C, indicating that there are two kinds of acid centers on the surface of the samples; the former is located in the low temperature zone (250 °C to 380 °C), corresponding to weak acid centers.^37–39^ The latter is located in the medium temperature area (380 °C to 500 °C), corresponding to medium acid centers.^40–42^

It can also be seen that the surface acidities are quite different for the catalysts prepared from different Ni–Al alloys. The weak and medium acid peak areas of the RNAA catalyst were lower than those of the others, indicating that the amount of acid centers available for adsorbing ammonia is low. RNAV, with higher weak and medium acid peak areas, demonstrated more acid centers.

**Fig. 10:**
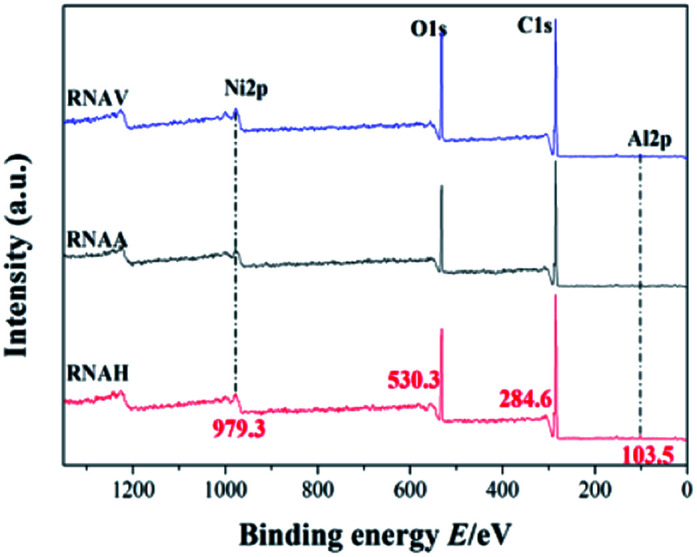
XPS profiles of the catalysts.

To research the valence state of each metal element on the catalyst surface, XPS analysis was carried out, as shown in Fig. 10. There are four peaks centered at 103.5, 284.6, 530.3 and 979.3 eV, attributed to Al 2p, C 1s, O 1s and Ni 2p, respectively.^43–45^ Nearly identical intensities of the Ni 2p peak at 979 eV for the different catalysts were detected, indicating that the RANEY®-Ni catalysts from the Ni–Al alloys prepared by different forming methods demonstrate almost the same interactions between Ni and the surrounding metal–Al.

**Fig. 11:**
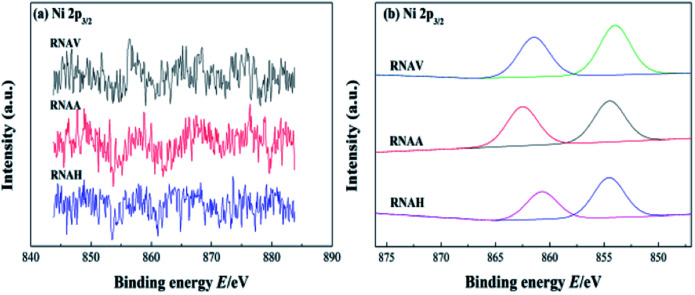
XPS profile after (a) and before (b) fitting of Ni 2p_3/2_.

Fig. 11(a) shows the XPS spectrum of Ni 2p_3/2_ of each catalyst. The peaks in the range of 840 to 890 eV were attributed to Ni 2p_3/2_; fitting of this peak was performed, and the fitting results are shown in Fig. 11(b) and Table 6.^46,47^

**Table 6:** Ni 2p_3/2_ binding energies of the catalysts

Samples	Ni 2p_3/2_-1 (±0.3 eV)	Ni 2p_3/2_-2 (±0.3 eV)
RNAV	861.4	854.0
RNAA	862.4	854.6
RNAH	860.6	854.5

There were two Ni 2p_3/2_ peaks, Ni 2p_3/2_-1 and Ni 2p_3/2_-2, in each catalyst. The former was located in the lower binding energy region (850 to 857 eV), and the latter was located in the higher region (857 to 865 eV). The binding energies of Ni 2p_3/2_-1 and Ni 2p_3/2_-2 for RNAA were 862.4 and 854.6 eV, respectively; these are higher than those of the other two catalysts, indicating that the active metal–Ni was more stable.

**Fig. 12:**
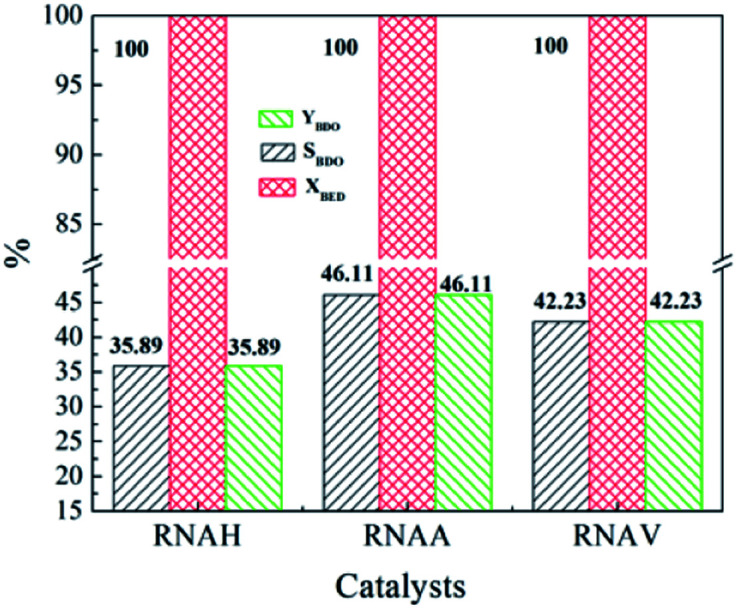
Catalytic properties of RANEY®-Ni catalysts for the hydrogenation of 1,4-butenediol. Reaction conditions: 5.0 MPa (pure H_2_), 393 K, 0.5 g catalyst, 30 mL 1,4-butenediol (35 wt% in water).

Fig. 12 shows the evaluation results of the performance of the prepared RANEY®-Ni catalysts in the hydrogenation of 1,4-butenediol (BED). It can be seen from Fig. 12 that the BED conversion of each catalyst was as high as 100%. Also, the selectivity of BDO obviously changed due to the different structure morphology of each catalyst. 1,4-Butenediol selectivity follows the following sequence: RNAA > RNAV > RNAH. RNAA showed better selectivity, with BED conversion of 100%, both BDO selectivity and BDO yield of 46.11%. According to catalyst characterization, the reason for the better performance of the RNAA sample may be related to the following factors: regular surface morphology, relatively complete leaching process (without Ni_2_Al_3_ and NiAl_3_ phases), high Ni/Al molar ratio and weak surface acidity. It is thought that the Ni/Al molar ratio, morphology and acidity strength are the most important factors. In addition, the selectivity for BDO of 46.11% is not beneficial for separating and purifying the product, and the catalyst preparation and reaction conditions should be further adjusted to increase the selectivity for the desired product.

**Fig. 13:**
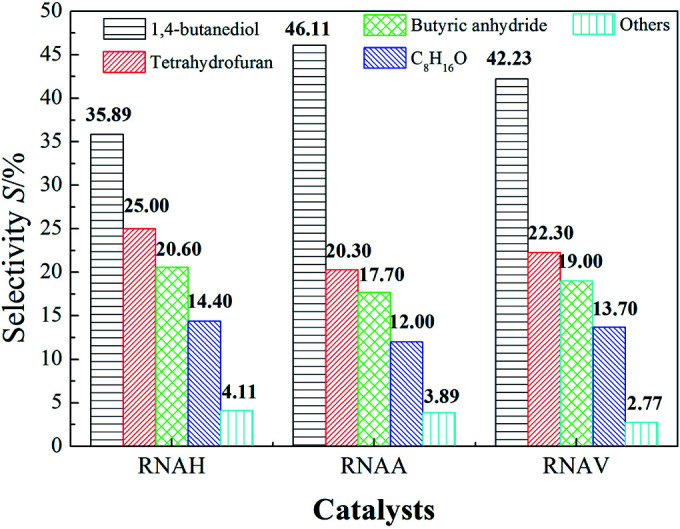
BED selectivities of the different catalysts. Reaction conditions: 5.0 MPa (pure H_2_), 393 K, 0.5 g catalyst, 30 mL 1,4-butenediol (35 wt% in water).

Fig. 13 shows the selectivity distributions of the hydrogenation products. The selectivities for 1,4-butanediol (BDO), 4-hydroxybutyraldehyde (HALD), tetrahydrofuran (THF), 1-ene-3-octanol (C_8_H_16_O) and other species, such as 2-hydroxytetrahydrofuran (HTHF) and 2-(4-hydroxybutoxy) tetrahydrofuran (HBOTHF), were compared. Each sample has good selectivity for 1,4-butanediol. It can also be seen from Fig. 13 that the selectivity of tetrahydrofuran was high (20%), next to BDO. Also the selectivities for 1,4-butanediol, tetrahydrofuran, butyric anhydride (BA) and 1-ene-3-octanol (C_8_H_16_O, EO) followed the order of BDO > THF > BA > EO.

**Fig. 14:**
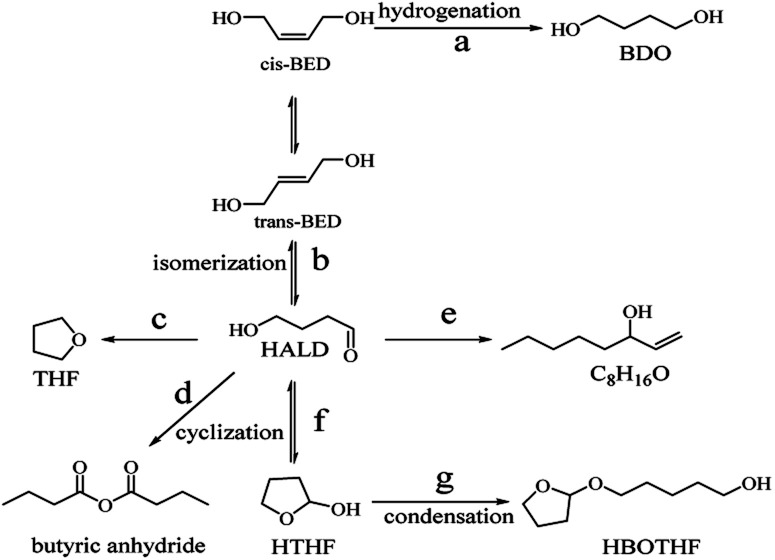
Proposed mechanism for the hydrogenation of 1,4-butenediol.

Based on previous reports^7,8^ and the current experimental results, a possible reaction pathway is proposed in Fig. 14. The raw material was a mixture of *cis*-1,4-butenediol and *trans*-1,4-butenediol, of which some was hydrogenated to obtain the target product 1,4- butanediol. The other part was isomerized to produce 4-hydroxybutyraldehyde. In addition, tetrahydrofuran, 1-ene-3-octanol (C_8_H_16_O) and butyric anhydride were obtained by a series of chemical reactions. There was also a very small amount of HALD to produce 1-hydroxyl cyclobutyl ether by cyclization and to form HBOTHF by a condensation reaction.

**Fig. 15:**
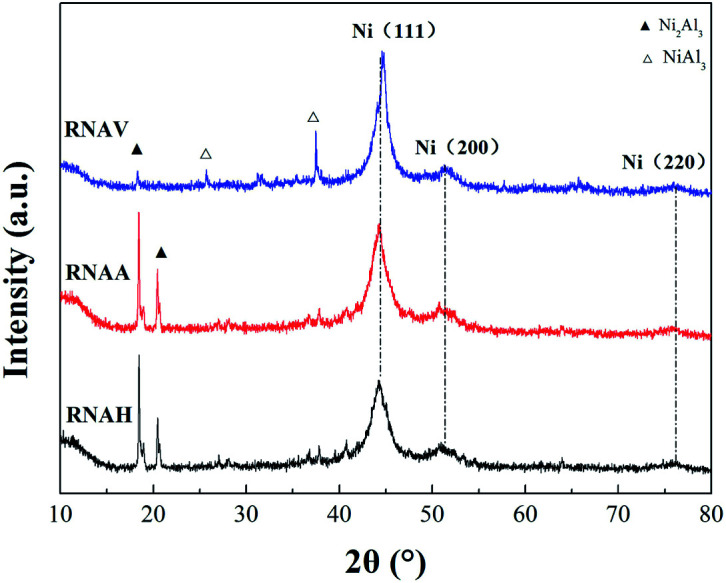
XRD diagrams of the samples after hydrogenation.

The used catalysts were subjected to XRD characterization, and the results are shown in Fig. 15. Diffraction peaks of the active component Ni still appear at 2*θ* = 44.1°, 51.3° and 76.3°. Compared with the fresh catalyst, the Ni(220) diffraction peaks detected in RNAA and RNAV at 2*θ* = 75.6° were weaker and more diffuse. This may be because the above two catalysts were subjected to high speed stirring for 3 h in the reactor, which may decrease the degree of Ni dispersion and destroy its crystal structure. For RNAH, Ni_2_Al_3_ diffraction peaks were detected at 2*θ* = 17.9° and 21.3°. The active component Ni and the structural promoter “Ni_2_Al_3_ crystal” did not significantly change compared with the fresh sample.^48,49^ For RNAA, the Ni_2_Al_3_ diffraction peaks were clearly detected at 2*θ* = 17.9° and 21.3°; this may also be derived from the stirring of the sample for 3 h at 120 °C and 400 rpm, which exposed the Ni_2_Al_3_ crystal and enabled it to be detected by XRD.

## Conclusions

The structure and properties of RANEY®-Ni catalysts obtained from different forming processes of Ni–Al alloy leached by 10 wt% NaOH solution were investigated. Based on previous reports^9–11^ and current experimental results, the properties of the materials obtained from various molding process and their physical and chemical properties showed great differences. It was found that the surface morphology, crystal structure, Ni/Al molar ratio and surface acidity of Ni–Al alloy and the corresponding catalysts varied greatly. Evaluation experiments showed that the RNAA catalyst from Ni–Al alloy by the atmospheric atomization method presented well catalytic performance with BED conversion of 100%, both BDO selectivity and BDO yield of 46.11%. The well performance might be related to the following factors: ① regular surface morphology; ② uniform crystal structure; ③ high Ni/Al ratio; and ④ weak surface acidity.

## Abbreviations

XRD: X-ray diffraction
SEM: Scanning electron microscopy
TEM: Transmission electron microscopy
NH_3_-TPD: NH_3_ temperature programmed desorption
XPS: X-ray photoelectron spectroscopy
EDX: Energy dispersive X-ray
BED: 1,4-Butenediol
BDO: 1,4-Butanediol
BET: Brunauer–Emmett–Teller
IUPAC: International Union of Pure and Applied Chemistry
BJH: Barrett–Joyner–Halenda
*L*
: Length, m
*t*
: Time, s
*m*
: Quality, g
*S*_BET_
: Specific surface area, m^2^ g^−1^
*D*
: Diameter, nm
*V*
: Volume per unit mass, mL g^−1^
*P*
: Pressure, MPa
*ν*
: Rotate speed, rpm

## Conflicts of interest

There are no conflicts to declare.

## Supplementary Material
